# Clinical and economic impact of medication administration errors among neonates in neonatal intensive care units

**DOI:** 10.1371/journal.pone.0305538

**Published:** 2024-07-11

**Authors:** Josephine Henry Basil, Nurul Ain Mohd Tahir, Chandini Menon Premakumar, Adliah Mhd Ali, Zamtira Seman, Shareena Ishak, Kwee Ching See, Maslina Mohamed, Khai Yin Lee, Nazedah Ain Ibrahim, Kokila Vani Jegatheesan, Noraida Mohamed Shah

**Affiliations:** 1 Centre for Quality Management of Medicines, Faculty of Pharmacy, Universiti Kebangsaan Malaysia, Kuala Lumpur, Malaysia; 2 Sector for Biostatistics & Data Repository, National Institutes of Health, Ministry of Health Malaysia, Shah Alam, Selangor, Malaysia; 3 Department of Pediatrics, Faculty of Medicine, Universiti Kebangsaan Malaysia, Kuala Lumpur, Malaysia; 4 Department of Pediatrics, Hospital Sungai Buloh, Ministry of Health Malaysia, Selangor, Malaysia; 5 Department of Pediatrics, Hospital Putrajaya, Ministry of Health Malaysia, Wilayah Persekutuan Putrajaya, Malaysia; 6 Department of Pediatrics, Faculty of Medicine, Universiti Pertahanan Nasional Malaysia, Kuala Lumpur, Malaysia; 7 Department of Pharmacy, Hospital Tunku Azizah, Ministry of Health Malaysia, Kuala Lumpur, Malaysia; 8 Department of Paediatrics, Hospital Cyberjaya, Ministry of Health Malaysia, Cyberjaya, Malaysia; University of Bahrain, EGYPT

## Abstract

Despite efforts in improving medication safety, medication administration errors are still common, resulting in significant clinical and economic impact. Studies conducted using a valid and reliable tool to assess clinical impact are lacking, and to the best of our knowledge, studies evaluating the economic impact of medication administration errors among neonates are not yet available. Therefore, this study aimed to determine the potential clinical and economic impact of medication administration errors in neonatal intensive care units and identify the factors associated with these errors. A national level, multi centre, prospective direct observational study was conducted in the neonatal intensive care units of five Malaysian public hospitals. The nurses preparing and administering the medications were directly observed. After the data were collected, two clinical pharmacists conducted independent assessments to identify errors. An expert panel of healthcare professionals assessed each medication administration error for its potential clinical and economic outcome. A validated visual analogue scale was used to ascertain the potential clinical outcome. The mean severity index for each error was subsequently calculated. The potential economic impact of each error was determined by averaging each expert’s input. Multinomial logistic regression and multiple linear regression were used to identify factors associated with the severity and cost of the errors, respectively. A total of 1,018 out of 1,288 (79.0%) errors were found to be potentially moderate in severity, while only 30 (2.3%) were found to be potentially severe. The potential economic impact was estimated at USD 27,452.10. Factors significantly associated with severe medication administration errors were the medications administered intravenously, the presence of high-alert medications, unavailability of a protocol, and younger neonates. Moreover, factors significantly associated with moderately severe errors were intravenous medication administration, younger neonates, and an increased number of medications administered. In the multiple linear regression analysis, the independent variables found to be significantly associated with cost were the intravenous route of administration and the use of high-alert medications. In conclusion, medication administration errors were judged to be mainly moderate in severity costing USD 14.04 (2.22–22.53) per error. This study revealed important insights and highlights the need to implement effective error reducing strategies to improve patient safety among neonates in the neonatal intensive care unit.

## Introduction

Globally, medication errors (MEs) are the leading cause of preventable patient harm [1]. They occur at various stages of the medication-use process, such as prescribing, transcribing and documenting, dispensing, administering, and monitoring [2]. Throughout these stages, errors were most common during administration. Among the 237 million MEs estimated to occur in England annually, the error rate during administration was found to be 54.4% [3]. In a systematic review of MEs in Southeast Asian countries, medication administration errors (MAEs) were also found to be the most common type of ME [4]. Furthermore, the likelihood of MAEs being intercepted was less likely than that of MEs being intercepted during other stages of the medication use process [5]. The fact that MAEs are the most common type of ME and are the least likely to be intercepted means that MAEs may cause more harm to patients and place a significant burden on the healthcare system [3, 6].

Although a plethora of studies have been conducted to investigate MEs, MEs remain a concern, especially among neonates [7]. One of the three key action areas identified by the World Health Organisation in its Third Global Patient Safety Challenge, which aims to globally reduce severe preventable harm as a result of MEs by 50% over a span of 5 years, is high-risk settings such as the Neonatal Intensive Care Unit (NICU) [1, 2]. In the neonatal population, MEs occur at different stages of the medication-use process, with percentages ranging from 14% to 74% for prescribing, 12% to 18.4% for transcription, 11.9% to 25% for dispensing, 31% to 63% for administering, and 1.4% for monitoring [5]. The prevalence of MAEs among neonates in the NICU has been reported to be as high as 94.9% [8]. Given that MAEs are so prevalent in the NICU, it is not surprising that the World Health Organisation has identified such high-risk situations as areas requiring much attention for the reduction of significant harm [2].

A study in the United Kingdom that evaluated almost 60,000 medication incidents reported to the National Patient Safety Agency found that MAEs led to the highest number of medication incidents resulting in severe patient harm or death [9]. In another study, it was estimated that in a hospital where 6 million doses of medications are administered annually, 4,000 patients are expected to be harmed [10]. In a recent systematic review conducted to examine the severity of MAEs among neonates, only three studies were found to have assessed severity using different assessment methods [8]. The challenge in classifying the severity of MAEs is reflected by the limited number of studies [11]. The assessment methods used for these studies were the National Coordinating Council for Medication Error Reporting and Prevention [12], a validated visual analogue scale [13] and a medication error review [14]. MAEs were harmful in 1% to 34.5% of the medication doses observed [15–17]. However, only one of the three studies used an appropriate validated assessment tool to classify potential harm as a result of MAEs in the Netherlands [15]. While the literature on the severity of MAEs among neonates is available [15–17], a valid, reliable, and appropriate tool to assess the severity of potential harm in a resource-restricted setting with a larger sample size will facilitate a better understanding of the clinical impact of MAEs.

From an economic standpoint, the World Health Organisation reported that MEs are estimated to cost USD 42 billion annually worldwide [1]. The cost of these preventable adverse drug events was estimated to range between USD 25 and 33 million in a study where 6 million doses of medications were administered annually [10]. In a retrospective review of claims made against the National Health Service in the United Kingdom over a span of 12 years by adult patients alleging that they have suffered from anaesthesia-related MAEs, MAEs were estimated to cost £4 283 677 [18]. To our knowledge, there is also no available literature on the economic impact of MAEs among neonates. Therefore, understanding the clinical and economic impact of MAEs on neonates in the NICU will enable the implementation of targeted interventions to reduce MAEs and highlight specific areas requiring further studies to reduce MAEs for policymakers.

Therefore, the purpose of this study was to determine the potential clinical and economic outcomes of identified MAEs and to identify the factors associated with them. The information obtained from this study will shed light on the consequences of MAEs among neonates in NICUs, enable the implementation of targeted interventions to reduce MAEs and highlight specific areas requiring further studies to reduce MAEs for policymakers.

## Materials and methods

### Design

A prospective direct observational study was conducted as a preliminary study to identify MAEs. A multidisciplinary expert panel consisting of three neonatologists, a paediatrician, a clinical pharmacist, and a senior nurse specialising in neonatology assessed each MAE for its clinical impact.

### Study setting

The national level, multi centre, direct observational study was conducted within the NICUs of five public hospitals affiliated with the Ministry of Health, Malaysia. Each hospital was purposefully selected from one of the five regions in Malaysia—Northern, Central, Southern, East Coast, and East Malaysia—to ensure representation of the two principal categories of public hospitals specialising in neonatology: major specialist hospitals and state hospitals. The NICUs in the selected hospitals were characterised by bed capacities ranging from 16 to 38. While variations existed in the number of nurses and patients across these hospitals, the duration of nurses’ duty hours was the same throughout all the shifts and hospitals. The general characteristics of the study sites are shown in Table 1.

**Table 1:** General characteristics of the study sites.

Characteristics	Study Site 1	Study Site 2	Study Site 3	Study Site 4	Study Site 5
Medication distribution system	Floor stock distribution system and unit-of-use packaging	Floor stock distribution system and unit-of-use packaging	Centralised intravenous admixture service for most antibiotics, floor stock distribution system and unit-of-use packaging	Floor stock distribution system and unit-of-use packaging	Floor stock distribution system and unit-of-use packaging
Type of prescription	Handwritten prescriptions used for preparation and administration of medications	Handwritten prescriptions used for preparation and administration of medications	Handwritten prescriptions used for preparation and administration of medications	Handwritten prescriptions used for preparation and administration of medications	Handwritten prescriptions used for preparation and administration of medications
Personnel involved in preparation and administration of medications	Preparation and administration of medications are performed by the respective nurse in charge of each patient	Preparation and administration of medications are performed by the respective nurse in charge of each patient	Preparation and administration of parenterals are performed by the medication nurse for the entire ward while preparation and administration of oral medications are performed by the respective nurse in charge of each patient	Preparation and administration of medications are performed by the respective nurse in charge of each patient	Preparation and administration of medications are performed by the respective nurse in charge of each patient
Number of beds	16 beds	14 beds	38 beds	24 beds	18 beds
Nurse-to-patient ratio	1:1 or 1:2	Ranged from 1:2 and 1:9	Ranged from 1:4 to 1:7	Ranged from 1:1 to 1:3	Ranged from 1:2 to 1:4

### Study sampling

Using a formula without finite population correction, a minimum sample size of 329 was required to achieve a 95% confidence level with a 5% margin of error [19]. The expected prevalence of MAEs in this study was based on the findings of another study with a similar social background and setting, i.e., 31% [20]. With an additional 20% dropout rate, the sample size required would be 395 drug administrations.

### Eligibility criteria

Medications prepared and administered by nurses via all routes were included in this study except:

1. Enteral feedings, parenteral nutrition, blood-derived products, medical gases and dietary supplement.
2. Medications not administered because the patient was absent during the medication rounds, lacked intravenous access or due to clinical reasons such as contraindications.
3. Rectal administrations, when neonatal-specific rectal dosage forms were unavailable, and paediatric rectal dosage forms were modified to lower doses.

### Data collection

The data were collected at these NICUs between April 2022 and March 2023. In this study, direct observations were conducted by two experienced clinical pharmacists, with a minimum of ten years of clinical experience, acting as observers. Before initiating observations, the observers received comprehensive training in the direct observation method of data collection, as described by Barker and McConnell [21]. Pilot observations were subsequently conducted to familiarise the observers with ward procedures and minimise Hawthorne effects. To further minimise the Hawthorne effect, nurses were informed that the study aimed to identify strategies for enhancing medication supply and distribution systems and understanding their constraints, rather than evaluating individual practices [22].

The NICUs were divided into sections based on ward configuration and patient acuity levels. Random selection using Excel®’s random number generator determined the nurse(s) responsible for drug preparation and administration in each section. Prior to the observation of drug preparation and administration, a written consent was obtained from participating nurses. Observers then shadowed consenting nurses closely during these processes. For ethical reasons, in the event of a potentially harmful MAE such as administering expired drugs [16] or tenfold overdoses [23], observers intervened in a non-judgmental manner. These errors, if left unaddressed, could have adverse consequences for patients and were thus included in the dataset.

To ensure data validity, clinical pharmacists at each study site, separate of the research team, observed 10% of drug preparations and administrations. They concurrently observed these drug preparations and administration with the observer but recoded them independently. Agreement between the findings of pharmacists and observers was essential to validate the data.

The error identification of each sample was independently assessed by two clinical pharmacists with at least six years’ of clinical experience, who were not involved in data collection. They independently assigned errors to the observed samples to minimise bias. Any discrepancies were addressed through a discussion with the research team to reach consensus.

**Fig 1:**
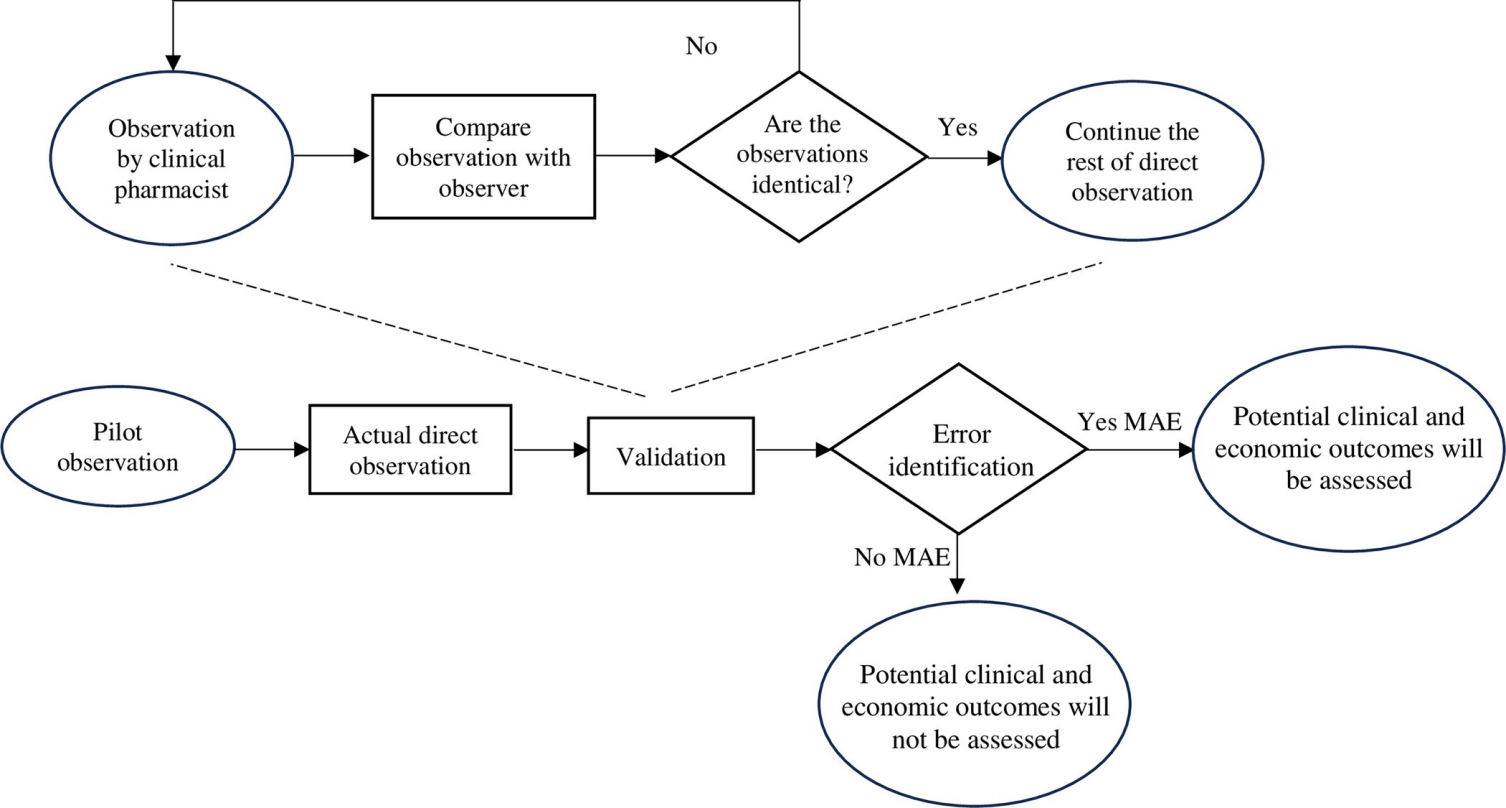
Study flowchart.

The comprehensive details of the data collection procedure are outlined in our published protocol for the development and validation of a risk prediction tool aimed at identifying neonates at risk of MAEs [24]. In summary, the flowchart for the direct observational study is shown below (Fig 1).

### Potential clinical outcome

The potential clinical outcome of the MAEs detected was determined using a validated visual analogue scale [13]. Dean and Barber recommended that at least four experienced healthcare professionals are required for a reliable, valid, and generalizable score of potential harm as a result of MAE [13]. The generalisability coefficient of an expert panel consisting of at least five judges was found to be 0.859. A coefficient of at least 0.8 indicates an acceptable level of reliability [13]. A clear relationship between potential and actual harm when using the validated visual analogue scale was established by Dean and Barber [13]. Hence, this is a suitable measurement scale for determining the severity of MAEs.

Generic anonymised case vignettes were created based on the detected MAEs from the direct observational study. One or more types of MAEs may occur simultaneously for each errored dose, and each type of MAE was represented by a case vignette. Patient-specific information such as the current weight and diagnosis was included in the anonymised case vignettes to better inform the panel when assessing the severity of the MAEs. The panels were briefed to assess the case vignettes based on the following assumptions: (1) the MAE reached the patient; (2) the MAE was a one-time event; (3) the other medications of the patient could be disregarded unless otherwise stated; and (4) the medication processes followed standard procedures of the ward unless otherwise described. MAEs occurring among neonates with the same diagnosis and within the same weight category for the same drug (extremely low birth weight < 1000 g, very low birth weight <1500 g, low birth weight < 2500 g) were combined into a single case vignette. The severity score for each of these combined case vignettes was then applied to the specific dose observed. Examples of case vignettes for each category of MAEs are provided in S1 Appendix. A similar method was employed in other studies [25, 26].

The expert panel was requested to individually and independently rate each case vignette using a visual analogue scale, which was defined as [13]:

- Minimal effect (0–2)—No anticipated lasting effects and only minimal patient discomfort.
- Moderate effects (3–6)—Likely to produce lasting effects and may interfere with treatment.
- Severe effects (7–10)—Likely cause life-threatening or lasting effects and could also result in death.

The expert panel was briefed by the principal investigator on the background of the study and the assumptions to be considered when rating each case vignette. The expert panel was allowed to ask questions about the drugs used in the case vignettes and access any references to assist them in rating the vignettes. However, they were not allowed to discuss or reveal their ratings to avoid influencing each other’s ratings.

### Potential economic outcome

Since the decision for a treatment to be rendered to treat MAEs is determined by the physician, only the neonatologists and the paediatrician of the expert panel were required to provide their opinion on the types of resources that would be expended in response to the MAE as a potential economic outcome. In this study, the cost of MAEs detected was defined as the additional costs incurred by hospitals to treat patients with MAEs. In Malaysia, healthcare services in the public sector are heavily subsidised by the government, with patients paying only nominal fees for both outpatient and inpatient services. The public healthcare sector caters to almost 70% of the population [27]. All costs considered in this study were derived from the perspective of the public healthcare provider. Only direct medical costs were considered in this study. Direct medical costs such as observations needed, drug therapy, diagnostic tests, laboratory tests, radiology tests, and additional days of hospitalisation were incurred as an immediate result of the MAE, while labour costs were the time required by the healthcare staff involved, such as the physician, nurse, and pharmacist, to address the consequences of the MAE [28]. The types of costs considered are important when quantifying the economic impact of MAEs, as the resources utilised for the treatment of these patients would not have been required had there been no MAEs.

The costs were derived based on the fee order [29–32], salary schedules [33–35], and local inventory drug price list. A consensus was reached among the experts for the amount of time spent by the physicians, nurses, and pharmacists treating the consequences of the MAEs. The labour costs of the healthcare team were then derived based on the salary schedules. Since the wages of neonatologists, general physicians, and pharmacists in the public sector depend on years of experience, these wages are based on the most common salary grade in Malaysia. This conservative approach was used to avoid inflation of the expected cost since the years of experience of these healthcare professionals varied greatly among the study sites. On the other hand, the wages for the nurses were based on the median nursing experience of 10 years among the nurses observed in the direct observational study.

In each of the case vignettes, the expert panel was asked to document the impact of the MAEs by choosing the consequences shown in Table 2.

**Table 2:** Categories of consequences due to MAEs [36].

Category of consequences	Type of resources
Observation	Number of additional physician (neonatologist or physician) encounters
	Type of vital signs monitoring initiated/increased
Drug therapy change	Initiation of a new drug therapy or a change in the current drug therapy such as increasing the duration of treatment
Diagnostic tests	Types of diagnostic, laboratory, radiology, or other diagnostic tests
Hospitalisation	Additional days of hospitalisation required
None	None

### Research variables and outcome measures

#### Study outcome

The outcome of interest in the direct observational study was the occurrence of MAEs among neonates in the NICU. MAE was defined as any deviation observed during the preparation or administration of medications from the medication order, hospital policy, or manufacturer’s instructions, as provided in the product leaflet [15]. The outcome of interest in this study was the potential clinical outcome, also known as the severity of MAEs detected, and the potential cost associated with these MAEs.

#### Independent variables

Various variables were identified through the literature and then reviewed by an expert panel consisting of a paediatrician with 14 years of clinical experience, a clinical pharmacist with 11 years of clinical experience, and a senior nurse who specialises in neonatology with 16 years of clinical experience. The identified variables were then categorised and defined in Table 3.

**Table 3:** Variables identified for statistical analyses.

Independent variables	Definition
*Administration-related variables*	
Route of administration	Route of administration for the medication administered to a patient (i.e., oral, intravenous, subcutaneous, intramuscular)
Number of medications administered	Number of medications prepared and administered by a nurse
*Working environment-related variables*	
Number of patients	Nurse to patient ratio
Working hours	Number of hours a nurse has worked up till the observation of the medications prepared and administered
Availability of protocol	Availability of a protocol related to the preparation and administration of medications at the study site
*Patient-related variables*	
Birth weight	The body weight of the neonate at birth
Gestational age	The gestational age of the neonate at birth
High alert medications	High-alert medication prescribed for the patient
Number of medications prescribed	Number of medications prescribed due for administration
*Individual-related variables*	
Experience at the study site	Total number of years at the NICU of the study site
Educational status	Level of education
Interruption and/or distraction	Interruption is defined as any stimuli causing the nurses to cease the preparation and administration of the medication temporarily, while distraction is defined as any stimuli that do not cause the nurse to cease the preparation and administration of the medication but cause the nurses to respond to the stimuli while continuing the drug preparation and administration.

### Data analysis

MAEs were categorised as 1 for observed doses with at least one error and 0 for observed doses with no errors. The overall MAE was computed by dividing the number of observations with at least one MAE by the sum of the observed doses and omitted doses.

## Ethical and research approvals

The study was conducted in accordance with the Declaration of Helsinki and approved by the Medical Research and Ethics Committee, Ministry of Health Malaysia (NMRR-21-1484-59494 [IIR]) and the Medical Ethics Committee, Universiti Kebangsaan Malaysia (JEP-2022-038). A written informed consent was obtained from the nurses prior data collection. We also obtained written approval and permission to conduct the study from the hospital director and the head of department of each study site.

### Potential clinical outcome

The ratings from the expert panel for the potential clinical outcome of MAEs were calculated based on the severity of MAEs, and their average was then categorised as mild (0–2), moderate (3–6), or severe (7–10). Univariate multinomial logistic regression was conducted to evaluate the associations between each independent variable. Mildly severe MAEs were assigned to the reference group. Variables with a p value of less than 0.05 according to the univariate analysis were included in the multivariate analysis using multinomial logistic regression. Variables with a p value of less than 0.05 were then selected using the backward stepwise method and retained in the final model. All the significant variables were assessed for multicollinearity, and variables with variance inflation factor values greater than 10 were excluded from the final model. Pearson chi-square statistics and classification results were assessed for model fitness. The strength of association for each of the independent variables was reported using crude and adjusted odds ratios (AORs). All the statistical tests were performed using IBM SPSS Statistics for Windows, version 28.0. Armonk, NY (IBM Corp.).

### Potential economic outcome

All cost estimates were provided in the local currency units of Ringgit Malaysia (MYR). It was then adjusted to the United States Dollar (USD) based on the World Bank’s growth domestic product per capita (PPP) of 1.58 [37]. This index reflects the units of the MYR needed, based on the country’s purchasing power, to purchase the same goods and services in the U.S. using USDs.

Simple linear regressions were conducted to examine the association between the cost of MAEs and individual independent variables. Variables with a p value of less than 0.05 were then included in the multiple linear regression analysis. The backward stepwise method was applied in the analysis for the final model, and variables were retained if their p values were less than 0.05. All the significant variables were assessed for multicollinearity and interactions. The assumptions of homoscedasticity and a normal distribution of variance for the final model were then assessed. The strength of association for each independent variable was reported using both crude and adjusted regression coefficients (B). All the statistical tests were performed using IBM SPSS Statistics for Windows, version 28.0. Armonk, NY (IBM Corp.).

## Results

### Direct observational study

The direct observational study included 170 patients, who were predominantly male (61.8%). The median (interquartile range, IQR) age was 35.0 weeks (IQR 9.0), while the median (IQR) birth weight was 1,950 g (IQR 1,665). The median (IQR) for the number of medications prescribed per patient was 2.0 (IQR 1.0).

A total of 1,093 medication doses were administered in the direct observational study. The rate of MAE was 68.0% (95% confidence interval (CI) 65.1%-70.7%). Among the 1,093 observed doses, 743 had at least one error, affecting 92.4% (157/170) of the neonates. Of the 743 observed doses with errors, a total of 1,288 types of MAEs were detected. The detailed MAE types detected are presented in Table 4.

**Table 4:** Frequency of errors according to the type of MAEs.

Types of MAEs	Total, n (%)
Wrong drug	1 (0.1)
Wrong dose	219 (17.0)
Wrong dosage-form	2 (0.2)
Deteriorated drug	173 (13.4)
Wrong drug-preparation	231 (17.9)
Omission	30 (2.3)
Wrong time	193 (15.0)
Wrong rate of administration	272 (21.1)
Administration without a medication order	1 (0.1)
Extra dose	1 (0.1)
Incompatibility	165 (12.8)
Total	1,288 (100.0)

### Characteristics of patients and nurses involved in the MAEs

A total of 157 patients experienced MAEs in the direct observational study. Ninety-seven of the patients were male. The median (IQR) gestational age was 35.0 weeks (IQR 8.8), while the median (IQR) birth weight was 1,930 g (IQR 1,660).

A total of 127 nurses were observed to have committed MAEs during the direct observational study. The average years of nursing experience at the NICU of the study site was 7.05 years (*SD* = 5.07), while the average overall years of nursing experience was 10.94 years (*SD* = 6.67). Half (66/127, 52.0%) of the nurses who committed MAEs were nurses with advanced diplomas in neonatology. The characteristics of the patients who experienced MAEs and the nurses who committed the MAEs are shown in Table 5.

**Table 5:** Characteristics of the neonates and nurses involved in the MAEs.

Characteristics	Categories	n (%)
*Patient*		
Gestational age	Extremely preterm (< 28 weeks)	19 (12.1)
	Very preterm (28 - <32 weeks)	40 (25.5)
	Moderate or late preterm (32 - <37 weeks)	41 (26.1)
	Term (> 37 weeks)	57 (36.3)
Birth weight	Extremely low birth weight (< 1000 g)	26 (16.6)
	Very low birth weight (< 1500 g)	31 (19.7)
	Low birth weight (< 2500 g)	45 (28.7)
	Normal birth weight (≥ 2500 g)	55 (35.0)
Sex	Male	97 (61.8)
	Female	60 (38.2)
Length of stay (days)	Median [IQR, (Min, max)]	6.0 [21.0, (3.0–24.0)]
*Nurse*		
Level of education	Diploma in Nursing	61 (48.0)
	Advanced diploma in neonatology	66 (52.0)
Overall nursing experience (years)	Mean (± SD)	10.94 (6.67)
Experience at the study site (years)	Mean (± SD)	7.05 (5.07)

### Potential clinical outcome

A total of 1,288 types of MAEs involving 66 drugs were included in this study. The expert panel reviewed 423 anonymised case vignettes to ascertain the potential clinical outcome of MAEs. The overall mean severity score in this study was 4.15, which is moderate for all MAEs detected. The majority of the errors, 79.0% (1,018), were found to be potentially moderate in severity, while 18.6% (240) of the errors were found to be potentially mild. Only 2.3% (30) of the errors were believed to be potentially severe. A detailed analysis of the types and severities of the errors is provided in Table 6, while examples of the errors according to their severity are provided in Table 7.

**Table 6:** Severity of MAEs (n = 1,288 errors out of 743 erroneous doses).

Category of MAEs	MAEs according to severity, n(%)			Total (%)
	Mild	Moderate	Severe	
Wrong drug	0	1 (0.1)	0	1 (0.1)
Wrong dose	57 (4.4)	155 (12.0)	7 (0.5)	219 (17.0)
Wrong dosage-form	0	2 (0.2)	0	2 (0.2)
Deteriorated drug	3 (0.2)	167 (13.0)	3 (0.2)	173 (13.4)
Wrong drug-preparation	87 (6.8)	144 (11.2)	0	231 (17.9)
Wrong time	78 (6.1)	111 (8.6)	4 (0.3)	193 (15.0)
Wrong rate of administration	13 (1.0)	248 (19.3)	11 (0.9)	272 (21.1)
Omission	2 (0.2)	23 (1.8)	5 (0.4)	30 (2.3)
Administration without a medication order	0	1 (0.1)	0	1 (0.1)
Extra dose	0	1 (0.1)	0	1 (0.1)
Incompatibility	0	165 (12.8)	0	165 (12.8)
Total	240 (18.6)	1,018 (79.0)	30 (2.3)	1,288 (100.0)

**Table 7:** Examples of MAEs according to severity.

Case vignette	Mean severity score	Potential outcome
*Severe*		
A neonate weighing 2,800 g was prescribed magnesium sulphate 800 mg stat for persistent pulmonary hypertension in newborns. However, magnesium sulphate 2,470 mg was administered instead.	8.83	Overdose may lead to unwanted adverse effects such as respiratory depression, respiratory paralysis, renal failure, coma, cardiac arrhythmias, and cardiac arrest.
A neonate weighing 670 g was prescribed noradrenaline as inotropic support. A dose was due at 10:00 a.m. However, it was only administered at 11:45 a.m.	8.17	Delayed administration of inotropes may be fatal.
A neonate weighing 761 g was prescribed sodium bicarbonate for severe hyperkalaemia. It was prescribed to be administered intravenously over 10 minutes. However, it was administered over 3 minutes.	7.50	Slow administration rates are recommended in neonates to minimise the possibility of producing hypernatremia, decreasing cerebrospinal fluid pressure, and inducing intracranial haemorrhage
*Moderate*		
A neonate weighing 880 g was prescribed levofloxacin for *Stenotrophomonas maltophilia* pneumonia. A single-use container of levofloxacin was opened on 20^th^ June at 10:00 a.m. A dose was withdrawn from this container and administered on 21^st^ June at 11:26 a.m.	6.83	Administration of a deteriorated drug could cause the efficacy of the drug to be compromised.
A neonate weighing 780 g was prescribed potassium chloride for hypokalaemia. A dose was due at 8:00 p.m. However, it was not administered.	6.67	The omission of a dose may lead to possible treatment failure
*Mild*		
A neonate weighing 3,110 g was prescribed calcium gluconate for hypocalcemia. A dose was withdrawn from the ampoule and then diluted in water for injection.	2.83	The use of water for injections as a diluent may be more likely to be associated with pain (possibly due to its hypotonicity).
A neonate weighing 895 gm was prescribed nystatin as prophylaxis for invasive candidiasis. A dose was due noon. However, the dose was administered at 1:10 p.m.	2.17	Delayed administration may cause the therapeutic failure of the drug.

According to the univariate analysis, factors significantly associated with moderately severe MAEs compared to mildly severe MAEs were the route of administration, number of medications administered, availability of protocol related to the preparation and administration of medications, gestational age, and nurses’ experience at the study site. While factors significantly associated with severe MAEs compared to mildly severe MAEs were route of administration, birth weight, gestational age, high alert medications, number of medications prescribed, and nurses’ experience at the study site. Details of the crude odds ratios (AORs), 95% confidence intervals (CIs), and p values are presented in Table 8.

**Table 8:** Univariate analysis of factors associated with the severity of MAEs.

Variable	Moderate		Severe	
	Crude OR (95% CI)	p value	Crude OR (95% CI)	p value
Route of administration				
Intravenous	2.95 (2.18–3.98)	< 0.001	3.51 (1.30–9.49)	0.013
Oral	1		1	
Number of medications administered	1.07 (1.03–1.12)	< 0.001	1.02 (0.92–1.13)	0.721
Number of patients	1.01 (0.96–1.05)	0.812	0.93 (0.80–1.09)	0.359
Working hours	0.98 (0.92–1.04)	0.455	0.87 (0.72–1.06)	0.177
Availability of protocol				
No	0.64 (0.46–0.88)	0.006	1.63 (0.60–4.45)	0.340
Yes	1		1	
Birth weight (kg)	0.90 (0.77–1.04)	0.158	0.52 (0.32–0.83)	0.006
Gestational age (weeks)	0.97 (0.94–0.99)	0.015	0.88 (0.81–0.96)	0.002
High alert medications				
Yes	1.23 (0.51–2.97)	0.653	7.80 (2.22–27.40)	0.001
No	1		1	
Number of medications prescribed	1.06 (0.98–1.14)	0.129	1.29 (1.09–1.52)	0.003
Experience (years)	1.03 (1.01–1.06)	0.025	1.06 (1.00–1.13)	0.060
Level of education				
Diploma in Nursing	0.90 (0.68–1.21)	0.497	1.65 (0.71–3.86)	0.248
Advanced Diploma in neonatology	1		1	
Interruption and/or distraction				
No	0.76 (0.49–1.18)	0.226	1.78 (0.40–7.87)	0.450
Yes	1		1	

*OR* odds ratio, *CI* confidence interval

After all the possible factors were adjusted as mentioned in the methods section, the probability of occurrence of moderately severe MAEs was found to be significantly greater for patients treated with intravenous medications (AOR = 2.84; 95% CI = 2.05–3.93; p<0.001), younger neonates (AOR = 0.95; 95% CI = 0.92–0.98; p = 0.001), and higher number of medications administered (AOR = 1.06; 95% CI = 1.02–1.11; p = 0.005). The significant risk factors for severe MAEs were intravenous medication (AOR = 5.82; 95% CI = 2.01–16.84; p = 0.001), high alert medications (AOR = 5.81; 95% CI = 1.58–21.33; p = 0.008), unavailability of a protocol related to the preparation and administration of medications (AOR = 3.04; 95% CI = 1.05–8.83; p = 0.041), and younger neonates (AOR = 0.85; 95% CI = 0.78–0.93; p<0.001). The adjusted odds ratios (AORs), 95% confidence intervals (CIs), and p values are shown in Table 9.

**Table 9:** Multivariable multinomial logistic regression for variables influencing the severity of MAEs.

Variable	Moderate		Severe	
	Adjusted OR (95% CI)	p value	Adjusted OR (95% CI)	p value
Route of administration				
Intravenous	2.84 (2.05–3.93)	< 0.001	5.82 (2.01–16.84)	0.001
Oral	1		1	
Number of medications administered	1.06 (1.02–1.11)	0.005	0.97 (0.86–1.10)	0.651
Availability of protocol				
No	0.87 (0.61–1.23)	0.434	3.04 (1.05–8.83)	0.041
Yes	1		1	
Gestational age (weeks)	0.95 (0.92–0.98)	0.001	0.85 (0.78–0.93)	< 0.001
High alert medications				
Yes	1.06 (0.43–2.64)	0.896	5.81 (1.58–21.33)	0.008
No	1		1	

*OR* odds ratio, *CI* confidence interval

The likelihood ratio chi-square test indicated that the full model had a good model fit, showing a significant improvement in fit compared to the intercept-only model (χ^2^ (10) = 90.68, p < 0.001). Overall, the accuracy of the model was 79.0%. Based on McFadden’s R^2^ statistics, the full model containing the predictors represented a 6% improvement in fit relative to the null model.

### Potential economic outcome

In this study, the potential cost of the 743 errored doses observed was estimated at MYR 43,664.16 (USD 27,452.10). Half of the potential cost, which amounted to MYR 21,861.84 (USD 13,749.58), was attributed to the additional observation needed as a result of the MAEs, while only 2.7% of the potential cost was attributed to the change in drug therapy (Table 10). The median (IQR) cost per MAE in this study was 22.19 (32.10) for MYR, which was 14.04 (2.22–22.53) for USD.

**Table 10:** Assessment of the potential cost of MAEs.

Categories of MAEs	The cost linked to additional observation (USD)	The cost linked to drug therapy change (USD)	Cost linked to additional diagnostic (USD)	Cost linked to prolonged hospitalisation (USD)	Total (USD)
Wrong drug	0	0	6.29	0	6.29
Wrong dose	2,854.24	110.41	1,802.67	2,216.98	6,984.30
Wrong dosage-form	32.31	0	23.58	0	55.89
Deteriorated drug	2,190.17	82.00	1,727.99	0	4,000.16
Wrong drug-preparation	1,649.54	7.31	360.85	1,698.11	3,715.81
Wrong time	939.53	2.01	827.83	0	1,769.37
Wrong rate of administration	4,640.14	0	1,410.38	141.51	6,192.03
Omission	405.80	79.82	172.96	1,273.58	1,932.16
Administration without a medication order	13.95	0	0	188.68	202.63
Extra dose	31.95	2.51	0	47.17	81.63
Incompatibility	991.95	459.70	1,069.18	0	2,520.83
Total	13,749.58	743.75	7,401.73	5,566.04	27,452.10

According to the univariate analysis, the route of administration and the use of high-alert medications were found to be associated with the cost of MAEs and were retained for the multiple linear analysis (p < 0.05) (Table 11). According to the multiple linear regression analysis, the regression model was found to be statistically significant (F = 8.022, p < 0.001; adjusted R^2^ = 0.011), and the statistically significant independent variables retained in the model were the intravenous route of administration (Adj. B = 9.08, 95% CI = 1.71–16.44; p = 0.016) and the presence of high-alert medications (Adj. B = 28.12, 95% CI = 10.59–45.64; p = 0.002) (Table 12).

**Table 11:** Simple linear regression for variables influencing the cost of MAEs.

Variables	B	SE	95% CI	*p* value
Route of administration (References: Oral)				
Intravenous	9.30	3.77	1.91, 16.68	0.014
Number of medications administered	0.02	0.40	- 0.76, 0.80	0.968
Number of patients	- 0.36	0.51	- 1.36, 0.65	0.487
Working hours	0.067	0.72	- 1.35, 1.48	0.928
Availability of protocol (References: Yes)				
No	5.90	3.42	- 0.81, 12.62	0.085
Gestational age	- 0.36	0.32	- 0.99, 0.27	0.261
Birth weight	- 1.16	1.71	- 5.02, 1.69	0.331
High alert medications (References: No)				
Yes	28.52	8.95	10.96, 46.07	0.001
Number of medications prescribed	0.69	0.81	- 0.90, 2.28	0.392
Experience (years)	0.35	0.27	- 0.18, 0.89	0.196
Level of education (References: Advanced diploma)				
Diploma	0.79	3.27	- 5.63, 7.20	0.809
Interruption and/or distraction (References: Yes)				
No	0.25	4.67	- 8.9, 9.41	0.957

*B* unstandardised regression coefficients, *SE* standard error, *CI* confidence interval

**Table 12:** Multiple linear regression for variables influencing the cost of MAEs.

Variables	Adj. B^a^	SE	95% CI	t-stat	*p* value
Route of administration (References: Oral)					
Intravenous	9.08	3.75	1.71, 16.44	2.42	0.016
High alert medication (References: No)					
Yes	28.12	8.93	10.59, 45.64	3.15	0.002

*B* unstandardised regression coefficients, *SE* standard error, *CI* confidence interval

^a^ Adjusted regression coefficients

R^2^ = 0.012; adjusted R^2^ = 0.011

## Discussion

The overall mean severity score in this study was 4.15 for all MAEs detected, with 85.5% of these MAEs being potentially moderate to severe. It was estimated that the MAEs detected could have potentially cost the Ministry of Health, Malaysia, close to MYR 45,000 (USD 28,000). Medications administered intravenously, the presence of high-alert medication, unavailability of a protocol related to the preparation and administration of medications, and younger neonates were significant factors associated with severe MAEs, while medications administered intravenously, an increased number of medications administered, and younger neonates were factors associated with moderately severe MAEs. Factors significantly associated with costlier MAEs were the intravenous route of administration and the presence of a high-alert medication.

The mean severity score and the proportion of moderately severe and severe MAEs in this study were greater than those in another study in the Netherlands conducted using a similar methodology, which reported that 57% and 1% of the MAEs were potentially moderately severe and severe MAEs, respectively [15]. The MAE in the study by Chedoe et al. [15] was 48.0%, whereas the error rate was 68.0% in our direct observational study. The difference in severity could be due to the difference in the error rate and the expert panel reviewing the MAEs detected. Despite having the same setting and methodology, the sample size in Chedoe et al was smaller than that in our study. Furthermore, instead of employing a minimum of four healthcare professionals to assess the severity of MAEs as recommended by Dean and Barber, an expert panel consisting of three healthcare professionals was used. Hence, a true representation of the population and the reliability of the severity scores may be questioned, and the results may not be extrapolated to the entire population.

A local study by Zainal et al reported that the median daily admission cost at two NICUs in Malaysia ranged from MYR 409 (USD 111) to MYR 496 (USD 135) [38]. The cost incurred for a median of two doses per drug administration round ranged between MYR 22.19 (USD 14.04) and MYR 110.95 (USD 70.22) per neonate in the direct observation study. Consequently, given the frequent drug administration rounds scheduled in a day, the potential cost incurred by MAEs alone could cost more than the median daily admission cost. The direct observational study was conducted at five study sites over a total of 60 days. Extrapolating these findings to a year suggests that the MAEs detected could approximately cost MYR 270,000 (USD 168,000) annually at these study sites. Although this sum may not seem significant, extending it nationwide to the 45 NICUs under the Ministry of Health, Malaysia, where healthcare is heavily subsidised and neonatal care is predominantly funded, the potential cost incurred by MAEs would be a significant burden. A recent study conducted in Denmark reported an annual incremental cost of €1,808,600 for an intervention implemented in the entire hospital, administering 2.3 million medications annually over a ten-year period [39]. Therefore, policymakers and stakeholders could consider redirecting the potential costs identified in this study towards the implementation of such error-reducing strategies.

Although the proportion of neonates potentially affected by severe MAEs is small, the number of neonates admitted to the NICU annually is approximately 3,400 across the five study sites, and the number of intravenous medications administered daily indicates that MAEs may be more common than expected and may cause significant patient harm [40]. In our study, drugs administered intravenously increased the risk of severe and moderately severe MAEs by 5.8 and 2.8 times, respectively. These were expected to cost MYR 9.08 (USD 5.75) more than drugs that are administered orally. The intravenous route of administration has been associated with an increased frequency of MAEs [8, 41]. This is mainly due to the increased opportunities for errors during administration as a result of the complex multistep preparation processes of the medications [5, 15]. According to a 5-year review of incident reports, the percentage of harmful intravenous-related MEs was greater than the percentage of all harmful errors combined [42]. Thus, it has been agreed universally that attention should be given to reducing intravenous MAEs, especially since they are associated with greater patient harm [41, 43]. Such strategies include implementing and enforcing protocols relating to the preparation and administration of intravenous medications and implementing a centralised intravenous admixture service [44, 45].

High-alert medications are medications with a heightened risk of causing significant patient harm when these medications are used erroneously [46]. Severe MAEs were almost 6-fold more likely to occur when high-alert medications were administered. In our study, significant linear relationships between the cost of MAEs and the use of high-alert medications (p = 0.004) were found, increasing the cost by MYR 28.12 (USD 17.80). A similar relationship between the severity of MAEs and high-alert medication use was found in a study by Sakowski et al. [47]. The use of these medications was also found to be more likely to result in harmful MEs among neonates in NICUs [48]. Therefore, various risk reduction strategies are recommended to reduce harm when high-alert medications are used during medication administration. Examples of these strategies include standardising the preparation and administration of these medications with the involvement of a clinical pharmacist [46, 49], implementing barcode medication administration safety and limiting the administration of high-alert medications to special authorised staff [46, 50], improving access to information about these medications [46] and enforcing double checking when administering medications [51].

In this study, we found that for each increase in the number of medications administered, the odds of having moderately severe MAEs increased by 6%. Studies have also suggested that an increase in nursing workload, indicated by the number of medications administered, is associated with errors such as omissions and situational violations, leading to severe MAEs and patient harm [52, 53]. Another factor significantly associated with both moderately severe and severe MAEs was gestational age. With an increase of one unit in gestational age (weeks), the odds of moderately severe and severe MAEs decreased by 5% and 15%, respectively. Extremely premature neonates are more susceptible to acute complications than their older and heavier counterparts [54, 55].

Our study identified the absence of a protocol related to the preparation and administration of medications as a significant factor associated with severe MAEs, with severe MAEs being three times more likely to occur in the absence of a protocol. This finding is consistent with previous studies that also found a significant association between absence of protocol and the occurrence of MAEs [56, 57]. Our results suggest that the absence of protocols may contribute to an increased risk of severe MAEs, highlighting the importance of implementing and adhering to established guidelines in healthcare settings. The significance of this finding is further emphasized by the World Health Organisation’s recognition of the importance of guidelines in enhancing healthcare professionals’ capacity and improving patient care quality [1]. By providing clear instructions and procedures, guidelines can help standardize medication administration practices, reduce variability, and minimize the likelihood of severe errors occurring [50].

Several predictors, while potentially holding practical importance in this study, were found to be solely significant in the univariate analysis. The inclusion of multiple variables in the multinomial logistic regressions revealed a more nuanced picture, where the significance of certain variables may be reverse when accounting for other predictors. Nevertheless, the clinical relevance of some of these predictors should be taken into consideration.

The relationship between nurses’ length of experience and MAEs in various acute-care settings has been extensively studied in the literature, with varying findings across different studies [58]. Most studies found that the greater nurse experience is associated with reduced occurrence of MAEs. This could be attributed to the advanced pharmacological knowledge typically possessed by experienced nurses, leading to enhanced medication safety [58]. While our study specifically investigated the severity of MAEs, our findings did not reveal a significant relationship between nurses’ experience and the occurrence of moderately severe and severe MAEs when compared to mild MAEs. Despite expectations that increased experience would correlate with decreased error severity, mirroring the observed trend in error occurrence, our results did not reveal this. One possible explanation to this discrepancy lies in the differing behaviors exhibited by junior and senior nurses in medication administration practices. Junior nurses, perhaps more cautious in adhering to medication orders, may demonstrate greater accuracy in medication administration. Conversely, senior nurses, while possessing extensive experience and knowledge, may not consistently adhere to protocols with the same precision, potentially leading to errors of greater severity [59]. This may contribute to the non-significant relationship observed between nurse experience and MAE severity in our study.

A 6-month study conducted in the United States identified that there was a curvilinear relationship between MAEs and the proportion of nurses with degree. While an increase in the number of nurses with degree was associated with a decrease in severe MAEs, no corresponding decrease was observed in non-severe MAEs [60]. The study also identified an optimal proportion of degree-prepared nurses at 54%, indicating that exceeding this percentage did not lead to further improvements; instead, the relationship became curvilinear. This finding may explain the higher incidence of moderately severe MAEs (79.0%) as compared to severe MAEs (2.3%), as 52.0% of the observed nurses held an advanced diploma in neonatology. Similar results were found in another direct observational study, where 68.6% of the detected MAEs were moderate, and 2.9% were severe [61].

Interestingly, interruptions were not found to be significantly associated with the severity of MAEs in our study, contrary to findings from previous research [62, 63]. For instance, prior study has reported a doubling of the estimated risk of majorly severe errors in the presence of four interruptions [63]. One plausible explanation for this contradicting finding lies in the differing prevalence of interruptions across studies. In our investigation, only 11.8% of nurses experienced interruptions or distractions during medication preparation and administration, compared to a markedly higher rate of 90.8% reported in another study [62]. Moreover, despite the implementation of interventions such as the ’Do Not Disturb’ vests and their inclusion as a parameter in the annual nursing audit in this study, adherence to wearing the safety vest remained low at 4.8%. This finding aligns with a recent study in France, which similarly found no impact of safety vest implementation on MAEs or interruption rates [64]. Therefore, we hypothesize that the significantly lower frequency of interruptions observed in our study may be attributed to the Hawthorn effect, whereby nurses modified their behaviour in response to being observed in a research setting [65].

### Strengths and limitations

This study has several limitations. Since the MAEs evaluated by the expert panel in this study were considered distinct events, the potential cumulative effects of multiple MAEs occurring simultaneously in a neonate were not included for review by the expert panel. The cost analysis conducted in this study may not be generalizable due to the variability in costs among the different geographic regions and payers. Direct nonmedical costs, indirect costs, and intangible costs were not included in this study. However, when considering these costs, the overall cost of MAEs would be much greater. Despite these limitations, this study is the largest direct observational study conducted among neonates in the NICU to assess the potential clinical outcome of the MAEs detected and the first to estimate the potential economic outcome of these MAEs.

### Recommendations for further research

Future research should focus on interventions overcoming the significant factors associated with the severity and economic impact of MAEs to improve patient safety.

### Implications for policy and practice

We anticipate that the insights attained from this study will assist policymakers and stakeholders in prioritising and implementing MAE-reducing strategies such as the implementation of a centralised intravenous admixture service and standardised national protocols related to the preparation and administration of medications by targeting the identified risk factors to prevent the occurrence of severe MAEs. Cost estimates from this study also provide a better understanding of the costs involved and further stress the need to identify cost-effective interventions to improve patient safety.

## Conclusion

The majority of MAEs detected were judged to be moderate in severity. The potential costs of the 743 errored doses observed were MYR 45,000 (USD 28,000) over 60 days, possibly leading to a significant economic burden. Factors associated with severe MAEs were the intravenous route of administration, the use of high-alert medications, the increased number of medications prescribed, and neonates with lower birth weights. Moreover, factors associated with moderately severe MAEs were intravenous medication and a greater number of medications prescribed. The use of high-alert medications and the intravenous route of administration were significantly associated with costlier MAEs. Understanding these factors is essential in implementing effective error-reducing strategies targeting the identified factors to improve patient safety among neonates in the NICU.

## Supporting information

S1 Dataset Dataset for the entire study. (XLSX)

S1 Appendix (DOCX)
