# Supplementary material for: Clinical and economic impact of medication administration errors among neonates in neonatal intensive care units
Source: PLoS One. 2024 Jul 11;19(7):e0305538. doi: 10.1371/journal.pone.0305538 (PMC11239004; doi:10.1371/journal.pone.0305538)
Supplement: S1 Appendix — (DOCX) [file pone.0305538.s002.docx]

**Clinical and economic impact of medication administration errors among neonates in neonatal intensive care units**

**PLOS One**

Josephine Henry Basil^1^, Nurul Ain Mohd Tahir^1^, Chandini Menon Premakumar^1^, Adliah Mhd Ali^1^, Zamtira Seman^2^, Shareena Ishak^3^, Kwee Ching SEE^4^, Maslina Mohamed^5^, Khai Yin Lee^6^, Nazedah Ain Ibrahim^7^, Kokila Vani Jegatheesan^8^, Noraida Mohamed Shah*^1^

1 Centre for Quality Management of Medicines, Faculty of Pharmacy, Universiti Kebangsaan Malaysia, Jalan Raja Muda Abdul Aziz, Kuala Lumpur, Malaysia.

2 Sector for Biostatistics & Data Repository, National Institutes of Health, Ministry of Health Malaysia, Shah Alam, Selangor, Malaysia.

3 Department of Pediatrics, Faculty of Medicine, Universiti Kebangsaan Malaysia, Jalan Yaacob Latif, Kuala Lumpur, Malaysia.

4 Department of Pediatrics, Hospital Sungai Buloh, Ministry of Health Malaysia, Selangor, Malaysia.

5 Department of Pediatrics, Hospital Putrajaya, Ministry of Health Malaysia, Wilayah Persekutuan Putrajaya, Malaysia.

6 Department of Pediatrics, Faculty of Medicine, Universiti Pertahanan Nasional Malaysia, Kuala Lumpur, Malaysia.

7 Department of Pharmacy, Hospital Tunku Azizah, Ministry of Health Malaysia, Kuala Lumpur, Malaysia.

8 Department of Paediatrics, Hospital Cyberjaya, Ministry of Health Malaysia, Cyberjaya, Malaysia.

*Corresponding Author:

**Name:** Noraida Mohamed Shah

**E-mail:** [noraida_mshah@ukm.edu.my](mailto:noraida_mshah@ukm.edu.my)

Appendix 1 - Examples of observed errors for each category of MAEs

**Appendix 1 Examples of observed errors for each category of MAEs**

| Category of MAEs | Examples of case vignettes |
| --- | --- |
| Wrong drug | A neonate weighing 1,459 g was prescribed ceftazidime for gram-negative rod sepsis (*Achromobacter spp*). However, cefepime was administered instead. |
| Wrong dose | A neonate weighing 880 g was prescribed amphotericin B 1 mg every 24 hours for candiduria. Amphotericin B 1.25 mg was administered instead. |
| Wrong dosage-form | A neonate weighing 880 g was prescribed intravenous omeprazole 1 mg for gastroesophageal reflux disease but a dose of oral omeprazole 1 mg was prepared and administered instead. |
| Deteriorated drug | A neonate weighing 880 g was prescribed ampicillin/sulbactam for nosocomial pneumonia (*Acinetobacter*). A vial of ampicillin/sulbactam was reconstituted on 20^th^ June at 8:00 p.m. and it was kept at room temperature. On 23^rd^ June, a dose was withdrawn and administered at 10:14 a.m. |
| Wrong drug-preparation | A neonate weighing 1,145 g was prescribed cloxacillin for nosocomial sepsis. A vial of cloxacillin (500 mg) was reconstituted with 5 ml of water for injection. A dose was withdrawn and administered without further dilution. |
| Wrong time | A neonate weighing 640 g was prescribed piperacillin/tazobactam for nosocomial pneumonia. A dose was due at 6:00 p.m. However, it was administered at 7:30 p.m. |
| Wrong rate of administration | A neonate weighing 920 g was prescribed azithromycin for presumed ureaplasma infection. It was administered by intravenous injection over 10 seconds. |
| Omission | A neonate weighing 1,100 g was prescribed cefepime for nosocomial sepsis. A dose was due at 8:00 p.m. However, it was not administered. |
| Administration without a medication order | A neonate weighing 1,850 g was previously prescribed caffeine 10 mg for apnoea of prematurity. This medication order was discontinued. However, caffeine was still administered. |
| Extra dose | A neonate weighing 3,140 g was prescribed clonazepam twice daily for dystonia. The administration schedule for clonazepam is 6:00 a.m. and 6:00 p.m. However, a dose was administered during the noon medication administration round at 1:01 p.m. |
| Incompatibility | A neonate weighing 1,420 g was prescribed benzylpenicillin and gentamicin for presumed sepsis. Gentamicin was administered immediately after the intermittent infusion of benzylpenicillin was completed without any flushing in between and at the same site. |
